# Examining the validity, reliability and feasibility of capturing children's physical literacy through games-based assessment in physical education

**DOI:** 10.3389/fspor.2023.1188364

**Published:** 2023-06-12

**Authors:** Brett Wilkie, Alastair Jordan, Jonathan Foulkes, Carl T. Woods, Keith Davids, James Rudd

**Affiliations:** ^1^ School of Science, Technology & Health, York St. John University, York, United Kingdom; ^2^ School of Sport & Exercise Sciences, Liverpool John Moores University, Liverpool, United Kingdom; ^3^ Institute for Health & Sport, Victoria University, Melbourne, Vic, Australia; ^4^ Sport and Human Performance Research Group, Sheffield Hallam University, Sheffield, United Kingdom; ^5^ Department of Teacher Education & Outdoor Studies, Norwegian School of Sports Sciences, Oslo, Norway; ^6^ Department of Sport, Food and Natural Sciences, Faculty of Education, Arts and Sports, Western Norway University of Applied Sciences, Sogndal, Norway

**Keywords:** assessment for / of learning, ecological dynamics, emergent behaviours, observational instrument, performance analysis, physical education

## Abstract

**Background:**

Observational tools can help refine practice design and guide the creation of effective learning environments. The intention of this study was to design and validate an observational instrument for assessing physical literacy that remains more faithful to the philosophically complex and holistic nature of the concept.

**Methods:**

Framed by concepts of ecological dynamics, the emergent games-based assessment tool enables capture of children's interactions with their environment, providing insight on the manifestation of physical literacy within physical education games. The design and validation of the instrument consisted of a multistage process: (1) design of the observational instrument and establishing face validity; (2) pilot observation study; (3) expert qualitative and quantitative review to establish content validity; (4) observation training; and (5), establishing observer reliability.

**Results:**

Following expert qualitative and quantitative evaluation, Aiken's *V* coefficient was used to determine content validity. Results achieved demanding levels of validity (*V* ≥ 0.78) for all retained measurement variables. Cohen's *κ* values for inter- and intra-observer reliability ranged from 0.331 to 1.00 and 0.552 to 1.00, generally reporting “substantial” agreement during inter-observer analysis and “substantial” to “almost perfect” agreement during intra-observer analysis.

**Conclusions:**

The final model of the emergent games-based assessment tool, with 9 ecological conceptualisations of behaviour, 15 measurement variables, and 44 categorical observational items was found to be valid and reliable, providing both educators and researchers with a useful mechanism to assess physical literacy during gameplay.

## Introduction

Observational instruments and methodologies are important to the study and wider development of physical education as a subject. Observational tools help to inform and guide the practice of teachers in their quest to design effective learning environments for children and youth. The last twenty years has seen significant investment in the development and application of systematic observational tools, enabling the identification of key teacher and child behaviours for effective learning and development during physical education and sport lessons (1–5). The purpose of systematic observation in pedagogy resides in efforts to improve the quality and value of educational intervention, supported by theoretically guided research that provides deep insights into professional behaviours and practices and why they should be encouraged or discouraged. Furthermore, systematic observations in physical education and sport provide authentic points of reference (demonstrated in context / in the practice environment), allowing for pragmatic understanding of what is typically considered to be an esoteric process to be developed (6). Overtime, these tools over time have replaced, or at least augmented other more traditional forms of evaluation (diagnostic “eyeballing”, subjective/anecdotal notetaking, completion of rating scales and checklists), in physical education assessments (7).

Physical literacy has rapidly permeated the practice and policy discourse among physical educators (8). Whitehead's (9) influential characterisation of physical literacy reimagined the concept in response to unease surrounding developments within physical education and concerns pertaining to physical inactivity patterns in the United Kingdom:

*A physically literate individual moves with poise, economy and confidence in a wide variety of physically challenging situations. The individual is perceptive in “reading” all aspects of the physical environment, anticipating movement needs or possibilities and responding appropriately to these, with intelligence and imagination. Physical literacy requires a holistic engagement that encompasses physical capacities embedded in perception, experience, memory, anticipation and decision-making*.

Commonly expressed themes within the literature highlight that physical literacy involves equal consideration of movement competence, attributes, behaviours, knowledge, understanding and the valuing of interactions with the physical world (8). Although the importance of accurate assessment of physical literacy has been established (10), there is need to develop tools that are both valid and capture the spirit of the whole construct successfully (11). Developing a framework for the assessment of a multifaceted phenomenon such as physical literacy is acknowledged as being difficult (12), and there remains tension between Whitehead's (9, 13, 14) characterisations of physical literacy and the manner in which it is typically assessed. Traditional assessments of physical literacy have been criticised, identifying that they are predisposed towards remaining reductionist in nature, product focussed, and tend to overly privilege movement competency components of the wider construct (15, 16). Recording the features of physical literacy that are not directly observable, such as the affective qualities of motivation or confidence, and the cognitive features of knowledge or reading the environment, is arguably more complex (12). In their major systematic review investigating measurement of physical literacy Edwards et al. (8) identified only three percent of the studies reviewed, combined all domains of physical literacy in their measurement of the construct. They went on to highlight the necessity to “assess beyond the constructs of physical proficiencies” (p. 660), calling for the adoption of a more holistic perspective. Although many existing instruments propose to provide a measure of physical literacy, the majority involve the evaluation of the cognitive, affective and psychomotor domains in isolation, often in decontextualized settings (17, 18). Separating the discrete domains of physical literacy during evaluation contradicts the holistic philosophical foundations of the construct (19). The disconnected performance of movement fails to effectively evaluate a child's capacity to alter, adapt, and combine movement skills according to performance needs. Flexibility and adaptation in movement, to reflect emerging environmental demands are important traits to advance competence and progress physical literacy (18). Previously, Wilkie et al. (20), developed a games-based assessment tool utilising an ecological dynamics framework providing a rationale that the cognitive, affective and psychomotor domains were observed through game play movement and captured using the emergent game-based assessment tool.

To help contextualise this study, we have summarised the key pillars of ecological dynamics (Wayfinding, Affordances and Attunement, Intentionality, and Functional Movement Skills) used in the design of the instrument, highlighting how they align to features of the physical literacy construct. Applied to a games-based context, wayfinding might reveal itself through the functionally effective solving of emergent, task-orientated movement challenges. For example, successfully negotiating emerging and disappearing gaps between competing players, or finding ways of distributing the ball favourably in regard to immediate task goals would be reflective of an individual's embodied knowledge and capacity to effectively navigate the performance environment. Since individuals perceive affordances in terms of their relevance and functionality, it is possible to develop an ecological understanding of cognition and decision making by integrating principles of affordances and attunement into the creation of observational assessments. From a physical literacy perspective, seeking and exploiting important information in the environment reflects embedded knowledge and understanding, as well as supporting the ability to effectively adapt to novel situations and settings. Contested and uncontested distribution, signalling for the ball, off-ball movements to break or establish dyadic stability, and effectively tracking play are examples of variables that would provide insight to an individual's attunement to affordances presented in small-sided games, revealing features of the cognitive domain of physical literacy.

Within an ecological dynamics framework it is possible to view motivation as the value or meaning given particular affordances, while intentionality captures the value and meaning of affordances during, and as a consequence of movement. Operationalising a person's intentionality as an expression of agency within an ecological dynamics conceptualisation of physical literacy is connected to the oscillating dynamics of the activity context and the perception of relevant information to enable exploitation of circumstances in an appealing manner. Coadaptive network formation when supporting play offensively, moderation of defensive pressure to close down, overload, or swarm opposition players, and defensive functionality in attempting or performing interceptions are examples of variables that provide insight to features of physical literacy's affective domain in small-sided games. Functional Movement Skills capture the qualities that allow individuals to negotiate their environment in the completion of intended task goals. Effective individual-environment interactions require holistic engagement whereby initial action results in further revelation of new affordances for competing and cooperating players and the individual themselves. The physically literate individual will demonstrate a fuller repertoire of emergent behaviours in pursuit of achieving task and performance goals, where cognition, perception, and action comprise the articulated functional movement observed. Heterogeneous or homogenous ball distribution; distribution distance, and direction, evidences features of physical literacy's movement competency domain in small-sided games.

This is the first tool of which we are aware that frames physical literacy assessment and evaluation through a “games environment” providing representative design (21) as well as better alignment to Whitehead's (9) original definition of physical literacy. There is however, more robust psychometric evaluation needed to explore the validity and reliability of the emergent games-based assessment tool before it should be used in education or intervention. Brewer and Jones (22) have previously noted that information related to validity and reliability of observational instruments was often inadequate and difficult to acquire. The development and subsequent validity and reliability evaluations of observational tools have increased more recently, both in terms of scope of application and the recognition of the process of assessing the psychometric properties of the tools developed. Numerous studies have emerged with the objective of validating observational instruments for performance in sports including, Climbing (23), Football (24–26), Tennis (27), Rugby Union (28), and Volleyball (29). Similarly in pedagogy, validation of observational instruments such as the Games Performance Assessment Instrument (30), which assesses skills, movements and cognitive decisions, Performance Assessment in Team Sports (31), assessing motor and tactical skills, and observing teaching physical education games (2), assessing lesson context and teacher interaction during physical education classes has been undertaken. Assessing the validity of an observational instrument is typically achieved through independent verification from experts on a topic and refers to the extent to which the methods employed adequately assesses what they claim to measure (32). Reliability describes the consistency within the employed analytical procedures across time or observers (33), typically achieved utilising some combination of test, retest protocol. Therefore, the aims of this paper are to build upon the theoretical model proposed by Wilkie et al. (20) that led to the development of the emergent games-based assessment tool, by establishing the validity and reliability of the psychometric properties of the assessment tool following a systematic process including face and content validity, combined with inter- and intra-observer reliability.

## Methods

To establish the validity and reliability of the emergent game-based assessment a multistage development process as outlined by Brewer and Jones (22), and recently adopted by Palao, Manzanares and Ortega (29),, Larkin, O'Connor and Williams (24), Torres-Luque et al. (27), and Fernandes et al. (25) was implemented. This process included the following stages: (1) review of the literature and consultation with experts in the field leading to designing the observational instrument and a category system establishing face validity; (2) pilot observation study; (3) expert qualitative and quantitative review to establish content validity; (4) observation training; and (5), establishing intra- and inter-observer reliability.

### Development and validation of the emergent games-based assessment tool

#### Stage 1: design of the observational instrument and establishing face validity

The initial design of the observational instrument has been detailed previously in Wilkie et al. (20). In brief, this involved first establishing performance indicators and operational definitions of variables to be used in the tool. The initial literature review commenced with an evaluation of offensive and defensive behaviour variables from the team games literature, framed within an ecological dynamics framework. Due to this being the first observational tool we are aware of that frames physical literacy assessment and evaluation through an ecological dynamics rationale, adapting an existing observational instrument as recommended by Brewer and Jones (22), was not possible. Consequently, the development of a theoretical framework to inform the observational instrument was necessary. This conceptual framework involved identifying key pillars of an ecological dynamics approach for the design of a game-based assessment. For this purpose, key ecological principles towards revealing physical literacy in games play were established. Building from this, a category system and their descriptions were introduced, discussed, trialled, and endorsed by academic and technical experts within the field (20). The intent was to make the category system within the instrument capable of capturing the actions that allow specification of key performance variables such as ball distribution, possession, turn-over, spatial-temporal interactions and tactical movements within small sided games typical of physical education settings to be recorded. This enabled ecological conceptualisations, measurement variables, and variable descriptions, to be finalised, framed around the key ecological principles of (1) Wayfinding; (2) Affordances and Attunement; (3) Intentionality; and (4) Functional Movement Skills. This process ensured the games-based assessment tool had categorical items that accurately represented the revelation of physical literacy through observed behaviours within small sided games, which, within the context of systematic observation would mean face validity can be assumed within a physical education environment.

#### Stage 2: pilot observation study

Before this validation and reliability study, an initial pilot feasibility test evaluated the emergent games-based assessment tool, with the objective of making modifications, where necessary. The original category system design was comprised of three ecological conceptualisations of behaviours, 18 measurement variables and 42 categorial observation items. The category system for the emergent game-based assessment tool was developed into an electronic coding module allowing digital video-based tagging (Dartfish Pro) to be completed. Feasibility pilot testing with 10-minutes of physical education games footage was completed by the lead author. This approach ensured all technical and tactical behaviours were identified within the observational instrument. During this process, numerous scenarios not included in the initial list of behaviour categories were identified, and following subsequent meetings with the academic and technical experts these further categories were defined and added to the coding instrument. The feasibility testing resulted in adding behaviours that previously had not been defined at a theoretical level, but which manifested during games play (e.g., a “hand-off” as a distribution category within offensive functionality, moving without obvious purpose within offensive and defensive intentionality). Similarly, at this stage of the design and validation process the reported frequencies across behaviour categories was reviewed, with the intent to use the data to demarcate, define, and confirm behaviour categories (e.g., within invitation for action, forward and penetrating pass direction categories were grouped to capture all “forward” distribution behaviours). At this point the observational manual was created in which the nine ecological conceptualisations of behaviours, 20 measurement variables and 61 categorial observation items (revised from the initial feasibility pilot) were named and defined. The outcomes related to the design of the observational instrument after the first two stages (development of the category system and pilot study) have been defined and described in detail previously (20). The processes reported in Stage 1 and 2 ensured the emergent games-based assessment tool had categorical items that accurately represented the revelation of physical literacy through observed performance behaviours within small-sided games, which, within the context of systematic observation would mean face validity can be assumed within the physical education environment.

#### Stage 3: expert qualitative and quantitative review to establish content validity

The content validity of an observational instrument scrutinises the relevance of the categories to the contextual content area under investigation. Consequently, a systematic observation instrument devised to record children's behaviours in small-sided games can be considered to have content validity if it thoroughly captures, or adequately samples the principal array of behaviours demonstrated by individuals with different physical literacy profiles within physical education environments. Brewer and Jones (22) identify that observed behaviours should be treated equally without privileging any particular behaviour, ensuring there are no behaviours that are incorrectly defined or neglected from the category system. Possessing a complete picture of the technical and tactical behaviours and interactions within children's small-sided games to reveal physical literacy will allow the emergent games-based assessment tool to have content validity, as it will mean the instrument effectively samples or fully captures the archetypal behaviours demonstrated by the individuals and the phenomenon under investigation.

In this study, evaluation of content validity ensured the emergent games-based assessment tool was representative of games play and effectively able to report on individuals as they navigated their environment / the technical and tactical elements of the games. To establish content validity, a qualitative and quantitative evaluation of the modified emergent games-based assessment tool was provided by expert practitioners (teachers and academics). The inclusion criteria established for forming the expert panel were: (1) post graduate qualifications in pedagogy or Sports Science; (2) to be active practitioners or researchers in the field of pedagogy, physical education, or Sports Science; (3) to hold senior leadership or academic positions within their educational institutions (e.g., head of Physical Education); (4) have a minimum of 10 years' experience within their field of expertise; and (5) to be able to demonstrate knowledge transfer within their field of expertise (publications, mentoring, or the delivery of professional development and training courses). Following similar approaches adopted within other sport observational instrument design and validation studies (24, 27, 28), the expert panel were required to provide opinions and feedback on: (1) degree of suitability in the definitions of behaviour variables used in the instrument (Adequacy); (2) the relevance of these behaviours in revealing physical literacy (Pertinence); (3) suitability of including the behaviour or variable as part of the observational instrument (Inclusion); and (4) the necessity to include behaviours otherwise not considered within the framework of the observational tool. The quantitative evaluation consisted of scoring 1–10 the adequacy and pertinence of behavioural variables measured in the observational tool and in the qualitative section, acknowledging the appropriateness of inclusion of the behaviour measured by responding “Yes” or “No”. By gathering the opinions of a range of experts we are able to establish that all elements of the game's environment, and the behaviours observed are appropriately considered when developing the instrument. The qualitative and quantitative evaluation data to establish validity was later calculated through Aiken's *V* coefficient (34, 35), to define criteria for acceptance, modification or elimination of items. Items of the emergent game-based assessment tool were evaluated firstly for pertinence and if deemed pertinent they were then evaluated for adequacy (definition).

#### Stage 4: observer training

Following the finalisation of the modified coding instrument an observer training programme was delivered. Rigorous observer training has been identified as particularly valuable in establishing the reliability and objectivity of an observation instrument (22). To this end, a multistage process of observer training, as outlined by van der Mars (36) was employed over a period of several weeks. The observer training was administered in a similar manner to that reported by Cushion et al. (1), Villarejo et al. (28), and Torres-Luque (27). For this research project two observers received training led by the principal investigator comprising of three, two-hour sessions, held over multiple days. The observers held post graduate qualifications in Physical Education and Sport Science, were active practitioners and researchers in the field of pedagogy and physical education, with over 10 years' experience within their field of expertise. Training focussed on: (1) orientation to the overall purpose of the instrument; (2) familiarisation of the categories; (3) using the coding timeline appropriately within Dartfish Pro; (4) embryonic coding of excerpts from physical education classes and games-play; and (5) the digital coding of one small sided, physical education game utilising the full emergent games-based assessment categorical instrument. The early stages of the training programme comprised of an introduction to the overarching purpose of the instrument, learning the behavioural classifications, and becoming familiar with the application of the temporal event-based nature of the observations within the coding timeline. The later phases included trialling the coding process with particular behaviours (e.g., single and multiple on / off-ball movement events), so that any misclassifications could be discussed and coding revised. Additional behaviours and extended passages of games-play were introduced as observers demonstrated consistency in classifying observations. Finally, a single small sided physical education game utilising the full emergent games-based assessment categorical instrument was coded. Embryonic and whole game electronic coding during stages 4 and 5 used recorded video playback of physical education games with frame-by-frame playback capabilities (1,080 hp, 60 Hz frame rate). Throughout the training process, observers and lead researcher reviewed and clarified any erroneous or unclassified behaviours to confirm understanding and interpretation of the instrument.

#### Stage 5: establishing observer reliability

To measure observer reliability, procedures outlined by Brewer and Jones (22) were followed. Observer reliability demonstrates the consistency or repeatability of an instrument for categorising behaviour. Therefore, to ensure the emergent games-based assessment tool is able to inform practice and contribute meaningfully to assessment and evaluation within a physical education environment, both intra- and inter-observer agreement should be established. This safeguards against variability within the results which may otherwise have ramifications upon the accurate interpretation of observed behaviours. Objectivity of the categorical system was evaluated to ensure that different observers could achieve a measure of agreement in their evaluations while observing the same behaviours. Inter-observer reliability was examined by reviewing the consistency between different observers when observing identical performances. Intra-observer reliability was explored by reviewing the measure of agreement between scores, when identical trials were evaluated by the same observer on two separate occasions. The intra- and inter-observer reliability was established using a 90-clip movie-test, similar to approaches adopted by Cushion et al. (1). The movie-test consisted of at least three examples of each ecological conceptualisation and all potential observational items from the emergent games-based assessment. Movie-test clips were purposefully selected with varying degrees of complexity demonstrated across the behaviours (e.g., single vs. multiple off-ball movements and shorter vs. longer temporal events). To ensure a comprehensive representation of the child-environment interactions possible, the game-play behaviours chosen for the movie-test included footage from three different primary schools, and comprised of mixed gender (3 Male, 3 Female), key stage 2 (aged 7–11 years), mixed physical literacy children. The physical literacy of children was calculated through a composite score of Working Memory (McClelland et al., 2014), Game Play Perception (Miller et al., 2019), and an assessment of fundamental movement skills using the Dragon Challenge (Stratton et al., 2015). To determine similarities between individual participants, a *kmeans* cluster approach was utilised (R Core Team 2019) to detect and organise data into a number of groups *via* the elbow method. This analysis enabled the grouping of individuals with similar levels of physical literacy into higher and lower-physical literacy categories. All movie-test clips were taken from a fixed, elevated, court side position. This perspective allowed for all participants, and features of the playing area to be captured within the field of view. The movie-test recordings were provided to the observers as an electronic video file (1080 hp, 60 Hz frame rate), with frame-by-frame playback options. Overall, 30 in-possession, 30 offensive off-ball, and 30 defensive off-ball events comprised the movie-test. A 90-clip movie-test was considered optimal as it provided acceptable variety in behaviours and enabled observers to complete the trial in a reasonable timeframe (90–120 min). The two trained observers conducted the electronic coding over a 14-day period, with a minimum of 7 days between the original and retest.

### Statistical analysis

All statistical analyses were performed in Microsoft® Excel® for Microsoft 365 (version 2301). The content validity of the quantitative evaluation of both pertinence and adequacy (definition) by the expert practitioners was determined by using Aiken's *V* coefficients (34). Aiken's *V* ascertains the magnitude to which experts deem items in a tool to represent and measure each construct (35). Aiken's *V* (34) was calculated using the formula:

ν=∑s/(c−1)

where the “*s*” was the score given by the expert practitioners minus the lowest value, “*c*” is the highest value and “*n*” was the number of expert practitioners. The Aiken's *V* coefficient ranges from 0 to 1, where 0 indicates lowest response from all experts (“not pertinent” or “poorly defined”), whereas 1 indicates highest response (“highly pertinent” or “very well defined”) from all experts (35). The central limit theorem and confidence levels were applied to the Aiken's *V* to set criteria for acceptance, modification or elimination of each item in the emergent games-based assessment tool. This process has been described in detail previously (37). In this study, an item was accepted if Aiken's *V* for pertinence was ≥0.78 (95% confidence level) and eliminated if Aiken's *V* was ≤0.77. Of the remaining items deemed pertinent, the adequacy (definition) of the items was modified if Aiken's *V* was ≤0.77 and eligible for modification between 0.78 and 0.86 (95%–99% confidence level).

Cohen's Kappa (*κ*) was used to measure the inter-rater reliability (between the observer responses) and intra-rater reliability (between original and retest responses) of the observers using emergent games-based assessment tool. Cohen's *κ* was the preferred measure of reliability with categorical data, since it accounts for the likelihood of agreement by chance (38) and was calculated using the formula:

$$\kappa =\frac{Po-Pe}{1-Pe}$$

where *Po* is the proportion of agreement, *Pe* is the proportion of agreement due to chance. Cohen's *κ* were interpreted as; ≤0 as no agreement, 0.01–0.20 as none to slight, 0.21–0.40 as fair, 0.41– 0.60 as moderate, 0.61–0.80 as substantial, and 0.81–1.00 as almost perfect agreement (38).

## Results

Expert qualitative and quantitative review to establish content validity was provided by 15 expert practitioners (7 teachers and 8 academics) with an average of 12.86 years' experience in their respective fields. All experts held post graduate qualifications in pedagogy or Sports Science, leadership or senior academic positions within their educational institutions, and were able to demonstrate knowledge transfer within their field of expertise. Aiken's *V* from the quantitative evaluation is shown in Table 1 drawing upon ecological conceptualisations defined in Wilkie et al. (20) to frame measurement categories.

**Table 1:** Content validity in the aspects of pertinence and adequacy (definition).

Measurement category linked to ecological conceptualisation	Measurement item	*Pertinence*			*Adequacy*		
			95% CI			95% CI	
		*V*	Low.	Upp.	*V*	Low.	Upp.
Passing (Offensive functionality)	Attempted passes	0.83	0.76	0.88	0.87	0.81	0.92
	Passing success	0.95	0.90	0.97	0.74^b^	0.77	0.89
Receiving (Offensive functionality)	Undisputed reception	0.87	0.81	0.92	0.86	0.69	0.83
	Disputed reception	0.89	0.83	0.93	0.74^b^	0.66	0.81
	Passing targets physical literacy	0.60^a^	0.52	0.68	–	–	–
Distribution technique (Offensive functionality)	Overhead pass	0.91	0.85	0.95	0.90	0.84	0.94
	Shoulder pass	0.88	0.82	0.92	0.84	0.77	0.89
	Chest pass	0.87	0.81	0.92	0.88	0.82	0.92
	Underarm pass	0.87	0.81	0.92	0.84	0.77	0.89
Distribution distance (Invitation for action)	Hand off	0.72^a^	0.64	0.79	–	–	–
	Short pass	0.85	0.78	0.90	0.86	0.79	0.91
	Medium pass	0.84	0.78	0.89	0.86	0.79	0.91
	Long pass	0.84	0.77	0.89	0.86	0.79	0.91
Distribution direction (Offensive invitation for action)	Penetrating pass	0.82	0.75	0.88	0.83	0.76	0.88
	Lateral pass	0.82	0.75	0.88	0.85	0.78	0.90
	Rearward pass	0.84	0.77	0.89	0.84	0.78	0.89
Offensive off-ball movement (Offensive intentionality)	Remains stationary on offence	0.79	0.72	0.85	0.71^b^	0.62	0.78
	Single offensive off-ball movement	0.77^a^	0.70	0.83	–	–	–
	Multiple offensive movements	0.82	0.75	0.88	0.68^b^	0.60	0.75
Use of space (Offensive spatial temporal interactions)	Finds space effectively	0.90	0.85	0.94	0.73^b^	0.65	0.79
	Moves into occupied space	0.82	0.75	0.88	0.83	0.76	0.88
	Moves into contested space	0.85	0.78	0.90	0.84	0.77	0.89
	Offensive Support	0.79	0.72	0.85	0.70^b^	0.62	0.77
Offensive awareness and interactions (Coadaptive networks)	Effectively tracks play on offence	0.86	0.79	0.91	0.81	0.74	0.86
Defensive off-ball movement (Defensive intentionality)	Remains stationary on defence	0.81	0.74	0.87	0.76^b^	0.68	0.83
	Single defensive off-ball movement	0.73^a^	0.65	0.79	–	–	–
	Multiple defensive movements	0.78	0.70	0.84	0.72^b^	0.64	0.79
Defender-attacker dyad (Defensive spatial temporal interactions)	Movement towards ball	0.90	0.84	0.94	0.81	0.74	0.87
	Swarms the ball	0.86	0.79	0.91	0.79	0.72	0.85
	Defensive marking	0.88	0.82	0.92	0.80	0.73	0.86
	No marking	0.74^a^	0.66	0.81	–	–	–
	Defensive pressure	0.79	0.71	0.84	0.73^b^	0.65	0.79
Defensive awareness (Coadaptive networks)	Effectively tracks play on defence	0.81	0.74	0.87	0.81	0.74	0.87
Interactions with ball (Defensive Functionality)	Defensive Intervention	0.90	0.84	0.94	0.84	0.77	0.89

*V*, Aiken's *V*; Low., lower limit; Upp., upper limit; CI, confidence interval; ^a^eliminated (*V* ≤ 0.77 in pertinence); ^b^ modified (*V* ≤ 0.77 in adequacy).

Expert review resulted in 5 measurement items being eliminated from the tool. Additionally, 9 of the remaining variables reported values lower than 0.78 in the aspects of Adequacy (definition). These item descriptions were revised and improved following the recommendations of the experts. Table 2 presents the qualitative evaluations issued by experts and the actions taken to improve the definitions used in the emergent game-based assessment tool.

**Table 2:** Qualitative expert evaluations issued and actions taken.

Measurement variable and qualitative evaluation	Action
**Passing success** E2: “pass contributed positively towards the team”s outcome” E3: “Receiving success to couple with the passing data” E8: “Rate of success (comparing successful passes with attempted passes)”	A clearer definition was determined for passing success. “*Successful distribution and reception of ball, contributing effectively to achieving game objectives”*
**Contested reception** E9: This can be very subjective measure, what is duress.	Item title and definition revised offering a clearer articulation of variable. “*Distribution was to a marked teammate resulting in a contested, but successful reception”.*
**Remains stationary (on offence and defence)** E1: “Movement rather than displacement to simplify definition” E9: “Consideration of a vertical component” E13: “Remaining stationary and taking steps are not the same”	The concepts of horizontal and vertical displacement were included and language was revised to assist clarity / understanding. “*No horizontal (e.g., stepping) or vertical (e.g., jumping) movements observed”*
**Multiple (offensive and defensive) movements** E1: “Change of direction or significant change in velocity” E9: “A player runs in one direction but then changes direction during the run without stopping vs. runs in one direction, stops/slows down and then changes direction” E14: “Whether the movement was done as one action, or multiple adjustments”	The concepts of changes in direction and changes in velocity were included to clarify the definition. “*Player demonstrates multiple significant changes of direction or changes in speed”*
**Finds space effectively** E2: “People can often find space, but it can often be too far away from the ball to receive a pass”. E9: “What (do) you describe as enough space” E15: “Recognition of invitation to move”	Item title and definition was revised. Distance from opposition players was specified to avoid subjectivity and acknowledgement of player location within the game's area relevant to task goals was added. “*Moves to, or occupies space (no other player within 1.5 m) appropriate to achieving game objectives”*
**Offensive support** E1: “Proximity to player in possession during offence” E9: “Definition relating to how many steps away a player would be from the player in possession might be better.”	Item title was revised and a clearer definition was determined incorporating specified distances to avoid subjectivity. “*Proximity to team mate in possession of the ball during offence (Close: within 1.5 m of player with ball; Nearby: 1.5–4.5 m away from player with ball; Distant: more than 4.5 m of player with ball)”*
**Defensive pressure** E1: “Appropriateness of defensive positioning”	Item title was revised and a clearer definition was determined representative of tactical intentionality incorporating specified distances to avoid subjectivity. “*Proximity to opposing player in possession of the ball during defence (Tight: within 1.5 m of player with ball and able to easily affect behaviours of the player in possession reflective of task goals; Loose: 1.5–4.5 m away from player with ball and able to influence gameplay behaviours reflective of task goals; Distant: more than 4.5 m away from player with ball exerting no or moderate effect on player in possession)”*

Aiken's *V* from the quantitative evaluation by the expert panel reported all variables not eliminated had an Aiken's *V* value >0.78 and were consequently considered relevant for inclusion (35). Consequently, the modified instrument (Table 3) included 15 measurement variables (6 related to on-ball offensive behaviours, 4 related to defensive off-ball behaviours, and 4 related to offensive off-ball behaviours during games play) and 44 categorical observational items for observation. All retained measurement variables scored highly (87%–100%) in expert review for suitability of including the behaviour as part of the final observational instrument.

**Table 3:** Emergent game-based assessment tool for the evaluation of physical literacy through an ecological dynamics framework.

Ecological conceptualisation of behaviour	Measurement variable	Categorical observation Item and description
Offensive functionality	Attempted Passes	Total number of observed pass attempts
	Passing	**Successful**—Distribution and reception of ball, contributing effectively to achieving game objectives. **Unsuccessful—**Failed distribution or reception of ball. **Successful shot**—“On” target. **Unsuccessful shot**—‘Off’ target. **Intercepted—**Interrupted progress, or course, of ball before arrival.
	Receiving	**Undisputed**—Distribution was to an unmarked teammate resulting in a successful reception. **Disputed**—Distribution was to a marked teammate resulting in a contested, but successful reception.
	Distribution technique	**Overhead Pass**—Throwing the ball with both hands—starting from behind the head and releasing the ball over or in front of the head. **Shoulder Pass**—Ball is held high (above the shoulder), the supporting hand is removed to propel the ball with a single hand releasing over or in front of the shoulder. **Chest Pass**—Using both hands, starting from their chest, arms are extended out propelling the ball forwards. **Underarm Pass**—One or two-handed pass that is initiated from below the waist.
Offensive invitation for action	Distribution distance	**Short pass**—Distribution involves ball travelling <1.5 m between teammates. **Medium pass**—Distribution involves ball travelling 1.5–4.5 m between teammates. **Long pass**—Distribution involves ball travelling >4.5 m between teammates.
	Distribution direction	**Penetrating pass**—Distribution is of a penetrative orientation (between 10 and 2 on a clock face where 12 on the clock face indicates the direction of opposition goal). **Lateral pass**—Distribution is of a lateral orientation (right lateral between 2 and 4, and left lateral between 8 and 10 on a clock face, where 12 on the clock face indicates the direction of opposition goal). **Rearwards pass**—Distribution is of a rearward orientation (between 4 and 8 on a clock face, where 12 on the clock face indicates the direction of opposition goal).
Offensive intentionality	Offensive off-ball movement	**Remains stationary on offence**—No horizontal (e.g., stepping) or vertical (e.g., jumping) movements observed. **Multiple off-ball movements**—Player demonstrates multiple significant changes of direction or changes in speed.
Offensive spatial temporal interactions	Use of space	**Finds space effectively**—Moves to, or occupies space (no other player within 1.5 m) appropriate to achieving game objectives. **Moves into occupied space**—Movement to an area occupied by a teammate (one or more cooperating players within 1.5 m). **Moves into contested space**—Movement to an area occupied by opposition player (one of more competing players within 1.5 m).
	Offensive support	Proximity to team mate in possession of the ball during offence. **Close**—Within 1.5 m of player with ball. **Nearby**—1.5–4.5 m away from player with ball. **Distant**—More than 4.5 m away from player with ball.
Offensive coadaptive networks	Offensive awareness and interactions	**Effectively tracks play**—Head and body orientation allows for effective scanning behaviour (following play, observing the ball, and positions of competing and cooperating players). **Does not track play**—Head and body orientation do not allow for effective scanning behaviour (following play, observing the ball, and positions of competing and cooperating players). **Calls or signals for ball**—Visual or verbal cue provided, indicating availability as distribution target.
Defensive intentionality	Defensive off-ball movement	**Remains stationary on defence**—No horizontal (e.g., stepping) or vertical (e.g., jumping) movements observed **Multiple off-ball movements**—Player demonstrates multiple significant changes of direction or changes in speed
Defensive spatial temporal interactions	Defender-attacker dyad	**Moves towards the ball**—Movement towards ball reducing ease (space and time) of player's subsequent actions. **Overloads the ball**—Movement towards the player with the ball affecting ease of opposition player's subsequent actions (second defender to close down the ball). **Swarms the ball**—Movement towards the player with the ball affecting ease of opposition player's subsequent actions (third defender to close down the ball). **Defensive marking**—Purposeful movement towards and subsequently occupying contested space attempting to mirror the movements of opposition player(s) without the ball. **Unpurposeful movement**—Movement is neither towards the ball or opposition player.
	Defensive pressure	Proximity to opposing player in possession of the ball during defence. **Tight**—Within 1.5 m of player with ball and able to easily affect behaviours of the player in possession reflective of task goals. **Loose**—1.5–4.5 m away from player with ball and able to influence gameplay behaviours reflective of task goals. **Distant**—More than 4.5 m away from player with ball exerting no or moderate effect on player in possession.
Defensive coadaptive networks	Defensive awareness and interactions	**Effectively tracks play**—Head and body orientation allows for effective scanning behaviour (following play, observing the ball, and positions of competing and cooperating players). **Does not track play**—Head and body orientation do not allow for effective scanning behaviour (following play, observing the ball, and positions of competing and cooperating players).
Defensive functionality	Defensive intervention	**Successful block or interception**—Player cuts out a pass or shot effecting progress, or course of ball through contact. **Attempted block or interception—**Player attempts to cut out a pass or shot. **No defensive intervention**—No attempt to effect progress, or course of ball through contact is made.

Movie-test coding typically took 90–120 min per test-retest coding exercise, resulting in 780 data entry points being recorded per observer during the movie-test. Cohen's *κ* following inter-observer and test-retest quantitative evaluation is shown in Table 4 drawing upon ecological conceptualisations defined in Wilkie et al. (20) to frame measurement categories.

**Table 4:** Cohen's *κ* inter- and intra-observer agreement following training.

Measurement category linked to ecological conceptualisation	Inter-observer reliability		Test-retest reliability	
	*κ*	Strength of agreement	κ	Strength of agreement
On-ball behaviours				
Attempted passes—Offensive functionality	1	Perfect	0.952	Almost Perfect
Passing—Offensive functionality	0.768	Substantial	0.701	Substantial
Receiving—Offensive functionality	0.724	Substantial	0.817	Almost Perfect
Distribution technique—Offensive functionality	0.944	Almost Perfect	1	Perfect
Distribution distance—Invitation for action	0.634	Substantial	0.841	Almost Perfect
Distribution direction—Invitation for action	0.941	Almost Perfect	0.942	Almost Perfect
Offensive off-ball behaviours				
Offensive off-ball movement—Offensive intentionality	0.590	Moderate	0.624	Substantial
(Initial) Use of space—Spatial temporal interactions	0.463	Moderate	0.602	Substantial
(Secondary) Use of space—Spatial temporal interactions	0.331	Fair	0.552	Moderate
(Initial) Offensive support—Spatial temporal interactions	0.650	Substantial	0.649	Substantial
(Secondary) Offensive support—Spatial temporal interactions	0.650	Substantial	0.874	Almost Perfect
Offensive awareness and interactions (Tracking play)—Coadaptive networks	0.634	Substantial	1	Perfect
Offensive awareness and interactions (Calls for ball)—Coadaptive networks	0.714	Substantial	0.682	Substantial
Target for pass—Coadaptive networks	0.911	Almost Perfect	0.911	Almost Perfect
Offensive positioning	0.918	Almost Perfect	0.799	Substantial
Defensive off-ball behaviours				
Defensive off-ball movement—Defensive intentionality	0.684	Substantial	0.832	Almost Perfect
(Initial) Defender-attacker dyad—Spatial Temporal Interactions (initial movement)	0.417	Moderate	0.603	Substantial
(Secondary) Defender-attacker dyad—Spatial Temporal Interactions (secondary movement)	0.389	Fair	0.652	Substantial
(Initial) Defensive pressure—Spatial Temporal Interactions	0.586	Moderate	0.802	Almost Perfect
(Secondary) Defensive pressure—Spatial Temporal Interactions	0.827	Almost Perfect	0.722	Substantial
Defensive awareness and interactions—Coadaptive networks	0.682	Substantial	0.661	Substantial
Defensive intervention—Defensive Functionality	0.732	Substantial	0.773	Substantial
Defensive positioning	0.770	Substantial	0.821	Almost Perfect

κ* = *Cohen's Kappa coefficient values.

The kappa values for inter- and intra-observer reliability ranged from 0.331 to 1.00 and 0.552 to 1.00, generally reporting substantial strength of agreement during inter-observer analysis and substantial to almost perfect strength of agreement during intra-observer analysis. Specifically, slight agreement was not noted for any criterion. Fair agreement was noted in spatial temporal interactions during attacking and defensive categories. Substantial, almost perfect, and perfect strength of agreement was reported for 85% of the measurement categories.

## Discussion

The study exemplified the multiple phases necessary to create, validate, and test the confidence of a games-based behavioural observational instrument that frames physical literacy assessment and evaluation through an ecological dynamics rationale. The intention of the study was to construct a scale for the assessment of physical literacy that remains more faithful to the philosophically complex and holistic nature of the concept, rather than conforming to the more linear and reductionist thinking prevalent in the physical literacy measurement field (39, 40). Content validity and reliability in this study have been demonstrated at a statistical level, establishing that the emergent games play assessment tool is able to measure the dynamic interactions observed between child and environment in small-sided, modified games.

The present study implemented a multiphase approach to validation following a systematic process adapted from Brewer and Jones (22). This validation process implemented comparable approaches to those previously adopted in research on the development of novel observation instruments in sport (24, 25, 27, 29). The systematic approach to establishing validity and reliability of the assessment tool commenced with a review of the literature and the development of a category system, with associated descriptions endorsed by academic and technical experts within the field (Stage 1). The tool was refined during piloting resulting in additional behaviours that previously had not been defined at a theoretical level, but which manifested during games play to be added, with frequency data used to demarcate and confirm revised behaviour categories (Stage 2).

### Expert contributions

A panel of experts was responsible for the validation of the revised instrument, reviewing the adequacy, pertinence, and inclusion of the observational measurement variables (Stage 3). The number of experts that participated in the current study was greater than the number in similar studies validating observational instruments in sport [e.g., (24, 25, 37, 41)]. The statistical values needed to ensure content validity was achieved by the emergent game-based assessment tool exceeded minimal levels (0.70) recommended by the literature (35), providing a more conservative level of acceptance for all measurement variables. In this study, responses from the expert panel revealed that the majority of variables had an Aiken's *V* greater than 0.78, and were consequently considered relevant (35). There were, however, some items of the emergent game-based assessment tool that reported Aiken's *V* values lower than 0.78 in aspects of Pertinence. These items were subsequently removed from the assessment tool. This process resulted in retaining the nine previously defined ecological conceptualisations of behaviour from the pilot, reducing the measurement variables by five to a total of 15, and reducing the categorical observational items by 17 to a total of 44 in the final modified version of the assessment tool. Additionally numerous items of the emergent game-based assessment tool reported values lower than 0.78 in the aspects of Adequacy (definition). Where these items were still deemed pertinent the descriptions were revised and refined following the qualitative commentary and recommendations of the experts. This process resulted in revising definitions of nine observational items (see Table 2). The revised item descriptions were mostly related to offensive and defensive intentionality, defining movements of individuals as they attempted to maintain a secure dyadic (1v1) system or disrupt attacker-defender stability. These actions are typically complex and require players to often execute several actions, sequenced together to achieve movement goals (42, 43). Ju et al. (26) identified the value of capturing these more complex interactions as they add greater transparency and insight to the overall evaluation. The other item descriptions revised at this stage related to spatial temporal interactions, which, while offering valid, reliable insight (44), have been identified as requiring appropriate observer training to ensure accurate representation of the behaviours (29). The complexity of the actions described necessitates the adoption of an unambiguous description to assist observer interpretation and accurate data collection. The revisions undertaken remained cognisant of this need throughout.

### Instrument objectivity

The level of intra- and inter-observer agreement reported (Stage 5) confirms that after familiarisation with the coding instrument and the completion of the observer training programme the instrument can be considered reliable. All aspects of the emergent game-based assessment tool reported “fair-perfect” strength of agreement for reliability, with 83% of behaviours reporting “substantial” or better scores. The scale of analysis used in the emergent games-based assessment (captured at an individual-environment interaction level), capturing key affordances that a child attunes to and how they are functionally playing the game, results in a complex observational process being undertaken. Since van der Mars (36) indicated agreement levels can be dependent upon the phenomenon under investigation and the complexity of instrument employed it is reasonable to expect variance between simpler and more complex environment interactions. Recurrent patterns of error or weaker agreement levels were noted in events where multiple behaviours emerged simultaneously, prolonged temporal events were observed, or spatial temporal evaluations were difficult to define with confidence. “Fair-moderate” strength of agreement was reported, for example, in spatial temporal interactions on both offence and defence, suggesting some reliability in the reporting of these behaviours. Consequently, these aspects of the emergent game-based assessment tool should be used with caution. Alternatively, there may need to be further consideration given to the observer training programme, with a greater focus on these elements to assure more reliable assessment and data reporting. The test-retest data for both offensive and defensive spatial temporal interactions returned improved (moderate-substantial) strength of agreement, indicating more consistent use and interpretation of the categorial observation items when employed by individual observers during the re-test. García-Ceberino et al. (37) and Ju et al. (26), have similarly reported variables establishing defensive and offensive behaviours in relation to defending and exploiting space as the least identifiable actions when quantifying performance. By comparison, shorter, single behaviour, limited locomotion examples, with easily defined spatial temporal interactions (e.g., offensive functionality) reported greater strength of agreement during the process of establishing observer reliability.

### Unique benefits of tool

There is an emerging rationale for research to look beyond established measures of physical proficiencies, or decontextualised, reductionist measures of the affective and cognitive domains and be creative in the development of nonconventional approaches to the evaluation of physical literacy (19, 39). Barnett et al. (40) have acknowledged that physical literacy assessments utilised in practice are typically considered to simultaneously reflect important elements of physical literacy, while concurrently not adequately representing the entirety of the construct. The emergent game-based assessment tool highlights the multidimensional nature of physical literacy, providing insight beyond singular domains of the concept. Opportunities to meaningfully interact with the environment are crucial to the phenomenological and existential foundations of the construct, with richer experiences facilitating the opportunity to understand and fulfil individual potential (8, 45). Analysing emergent behaviours within physical education games play, where decisions are expressed as actions allows for a more holistic, embedded evaluation of physical literacy to be undertaken in more authentic settings. Knowing through movement is articulated through perception, action and skilled intentionality, helping establish an understanding of how individuals choose to act in specific circumstance. Knowledgeable performers should be able to respond appropriately, using intelligence and imagination to interact with events, objects and others in the environment. The emergent game-based assessment tool captures cognition that is embedded and embodied, as a consequence of perceiving affordances related to temporal, spatial and amplitude features of the surrounding environment to achieve success in designated tasks (46). This evidence-based conceptualisation aligns with insights of Turvey and Carello (47), from the perspective of ecological realism, who argued that cognition may be considered as the coordination of intentional interactions with the environment. The emergent game-based assessment tool, in remaining more loyal to the complex and holistic nature of the concept, is able to simultaneously provide insight to numerous domains of the construct.

Additionally, the feasibility of primary school physical education teachers, who are already balancing a burdensome work schedule being able to undertake appropriate assessments for whole classes remains unclear (48). Essiet et al. (49) nonetheless suggested that comprehensive evaluation of physical literacy is possible within the constraints of the professional teaching role. Embedding physical literacy assessment, using the emergent game-based assessment tool as part of normal physical education curricula mitigates against issues such as the additional time, equipment, and resources necessary to undertake alternative assessments. Such an approach would align to the emerging trend to adopt more ecological, embedded, qualitative assessment approaches in school settings (19, 50, 51). The emergent games-based assessment has the potential to chart growth over time, supporting assessment *of*, and *for*, learning in physical education to become a realistic possibility, due to how easily it can be embedded into existing practice employing games as part of the curriculum. Charting progress allows for the consideration of physical literacy at an individual level, recognising it is unique to the person, their endowment, and organism constraints (52). Monitoring progress in physical literacy is encouraged, as it can assist practitioners in the development of effective pedagogies (48). Assessment *for* learning in this manner is conducted principally to support and promote students' learning, creating a virtuous circle of teaching and student progression informing future steps in curriculum delivery. By comparison, assessment *of* learning enables the evaluation of the effectiveness of a learning process, typically associated with accountability, certification, or intervention evaluation. Linked to the key stage two National Curriculum for physical education (53), the emergent games-based assessment provides opportunities for assessment *of*, and *for*, learning. The National Curriculum identifies movement skills in isolation and in combination, the application of basic principles of attacking and defending, and the ability to compare current performance to past performance, recognising their own success as foci for development. The emergent games-based assessment can support practitioner evaluation of physical literacy in a formative, ipsative manner supporting assessment *for* learning, or alternatively in a broader, summative fashion, supporting assessment *of* learning, within the wider framework of the National Curriculum.

### Limitations

Some of the limitations noted are not dissimilar to those previously reported in other studies of observational research tools and include issues such as analysis complexity due to detailed categorisation (44), the labour-intensive nature of coding (25), potential confusion and subjectivity in accurately reporting behaviours (54) and the unpredictability of an individual's environmental interactions and the potential for player congestion (44). Nonetheless, this study evidences that the emergent game-based assessment tool has the potential to be highly effective following familiarisation and training. Future research should look to establish effectiveness and feasibility of use by practitioners in physical education settings, as well as considering the application of key ecological principles towards the manifestation of physical literacy in other physical activities and sports.

## Conclusions

This study found the ecological emergent games-based assessment tool to be valid and reliable, providing both educators and researchers with a useful mechanism to assess physical literacy during small-sided modified games. This is the first tool, of which we are aware, that frames physical literacy assessment and evaluation through an ecological dynamics rationale. Tapping into children's knowledge of the environment, revealed through children's perception of affordances, their intentionality, and how they functionally move to effectively wayfind through performance environments provides novel insight into how physical literacy manifests itself through embodied individual-environment interactions. The emergent games-based assessment values the holistic and multidimensional orientation of the physical literacy construct and has the potential to chart growth over time, support assessment for learning, and enable educators to better understand children's knowledge of the environment and how physical literacy reveals itself through embedded actions. Such insight could develop deeper understandings of the physical literacy construct, in addition to supporting a fulfilling physical literacy journey.

## Data Availability

The raw data supporting the conclusions of this article will be made available by the authors, without undue reservation.
